# Finding a Toll on the Route: The Fate of Osteoclast Progenitors After Toll-Like Receptor Activation

**DOI:** 10.3389/fimmu.2019.01663

**Published:** 2019-07-17

**Authors:** Pedro P. C. Souza, Ulf H. Lerner

**Affiliations:** ^1^Faculty of Dentistry, Federal University of Goiás, Goiânia, Brazil; ^2^Centre for Bone and Arthritis Research at Department of Internal Medicine and Clinical Nutrition, Institute for Medicine, Sahlgrenska Academy at University of Gothenburg, Gothenburg, Sweden

**Keywords:** toll-like receptors, osteoclast, lipopolysaccharide, RANKL, bone resorption

## Abstract

M-CSF and RANKL are two crucial cytokines stimulating differentiation of mature, bone resorbing, multinucleated osteoclasts from mononucleated progenitor cells in the monocyte/macrophage lineage. In addition to the receptors for M-CSF and RANKL, osteoclast progenitor cells express receptors for several other pro- and anti-osteoclastogenic cytokines, which also regulate osteoclast formation by affecting signaling downstream M-CSF and RANKL receptors. Similar to many other cells originating from myeloid hematopoetic stem cells, also osteoclast progenitors express toll-like receptors (TLRs). Nine murine TLRs are expressed in the progenitors and all, with the exception of TLR2 and TLR4, are downregulated during osteoclastogenesis. Activation of TLR2, TLR4, and TLR9, but not TLR5, in osteoclast progenitors stimulated with M-CSF and RANKL arrests differentiation along the osteoclastic lineage and keeps the cells at a macrophage stage. When the progenitors are primed with M-CSF/RANKL and then stimulated with agonists for TLR2, TLR4, or TLR9 in the presence of M-CSF, but in the absence of RANKL, the cells differentiate to mature, bone resorbing osteoclasts. TLR 2, 4, 5, and 9 are also expressed on osteoblasts and their activation increases osteoclast differentiation by an indirect mechanism through stimulation of RANKL. In mice, treatment with agonists for TLR2, 4, and 5 results in osteoclast formation and extensive bone loss. It remains to be shown the relative importance of inhibitory and stimulatory effects by TLRs on osteoclast progenitors and the role of RANKL produced by TLR stimulated osteoblasts, for the bone resorbing effects *in vivo*.

## Introduction

Human body is constantly exposed to microorganisms. In addition to our own cells, humans host a vast community of microbes, with an estimation of the number of bacteria exceeding the number of host cells by a factor of 1.3 (1, 2). The majority of these microorganisms populate the gastrointestinal tract and regulate processing and absorption of nutrients and vitamin biosynthesis, which impacts the development and remodeling of multiple organs, including bone (3). Recently, it has been demonstrated that disturbances in normal microbial population are associated with effects on bone, not only due to impaired uptake of nutrients, but also due to the activation of pattern-recognition receptors (PRRs) expressed in immune cells by microbe-associated molecular patterns (MAMPs) released by microorganisms (4–6). The intestinal microbiota modulates unexpected events distant to the mucosal surface, such as sex steroid deficiency induced bone loss (7). In contrast to wild type mice, sex steroid-depleted germ-free mice, fail to increase osteoclastogenic cytokines and, consequently, bone resorption is not increased and bone mass is preserved. Microbial recolonization restores the capacity of sex steroid depletion to induce trabecular bone loss. Interestingly, a shift in the normal microbial population by supplementation with probiotics protects mice from sex steroid depletion-induced bone loss. Corroborating these observations in mice, a double blind, placebo-controlled clinical trial demonstrated that daily intake of *Lactobacillus reuteri* for 12 months reduces the loss of volumetric bone mineral density (BMD) in 75–80 year old women who had low BMD (8).

The effect of MAMPs in bone metabolism becomes evident in infectious diseases close to the skeleton. In periodontitis, a highly prevalent inflammatory disease afflicting more than two thirds of Americans aging more than 65 years, bone loss is clinically observed due to infection by pathogenic bacteria and their recognition by the host immune system (9). Bacteria-induced bone loss is also involved in the pathogenesis of osteomyelitis (10). Bone resorption due to excessive osteoclast formation is also observed in *Staphylococcus aureus* septic arthritis (11, 12), an uncommon, but not rare disease affecting 2–10 patients of 100,000 in the general population (13). Not only MAMPs can activate PRRs since these receptors recognize also endogenously produced molecules such as danger-associated molecular patterns (DAMPs).

To study the interactions between bone and immune cells, the field of osteoimmunology emerged almost 50 years ago. In 1970, a breakthrough publication by Haussman et al. reported that endotoxin from the microorganism commonly found in the gingival sulcus, *Bacteroides melaninogenicus*, was as potent as parathyroid hormone in its ability to induce osteoclastogenesis and bone resorption (14). Two years later, Horton et al. described a factor released by leucocytes exposed to dental plaque that stimulated bone resorption in organ cultures of fetal rat radii by increasing the number of osteoclasts (15). These were the first evidence that bacterial components could indirectly affect bone metabolism through activation of inflammatory cells. Since then, the mechanisms underlying the interactions between inflammatory cells and bone cells have been extensively studied, particularly the role of cytokines in inflammatory bone loss (16).

A great advance in the field of osteoimmunology became possible after the breakthrough discoveries in late 1990's related to the characterization of Toll-like receptors. Toll protein, primarily related to dorso-ventral embryo patterning of *Drosophila melanogaster* (17), was identified in 1996 as a critical molecule for the response against the fungus *Aspergillus fumigatus* (18). Its homologous in humans, once called hToll and now Toll-like receptor 4 (TLR4), was shown 1 year later to be linked also to cytokine production in human monocytes (19). The identification of a mutation in the *Tlr4* gene in mice that render them resistant to endotoxin confirmed the participation of TLRs in innate immunity (20).

Not surprisingly, osteoclasts, which are derived from the hematopoietic stem cells, express TLRs and respond to MAMPs (21). Thus, the effect of TLR activation in osteoclasts and their precursors is an important aspect in the pathogenesis of inflammation-induced bone remodeling. In this review, we aim to dissect the molecular mechanisms underlying the effects of TLRs in osteoclast biology.

## Osteoclasts, Bone Cells Emerging From the Immune System

The clinical observation of local and systemic bone loss in a variety of inflammatory diseases demonstrates the influence of inflammation on bone metabolism (22). These diseases include rheumatoid arthritis, psoariatric arthritis, ankylosing spondylitis, septic arthritis, periodontitis, inflammatory bowel disease, osteomyelitis and loosening of joint prosthesis, and dental implants. The effect by the inflammatory process is most often locally in joints or jaw bones, but rheumatoid arthritis and inflammatory bowel disease also cause systemic bone loss, so called secondary osteoporosis. In periodontitis, failed dental implants and septic arthritis, bone loss is associated with infections by bacteria known to activate TLRs, but these receptors can also be activated by endogenous substances produced by cells in the inflamed joint in patients with rheumatoid arthritis. The expansion of the knowledge in the osteoimmunology field has helped us to understand how bacteria and tissue-produced ligands can regulate bone remodeling by activating TLRs.

Mouse monocytes and macrophages from different origins, such as bone marrow, spleen, thymus and peripheral blood, are capable of differentiating to mature osteoclasts when co-cultured with stromal cells in the presence of 1,25-dihydroxyvitamin D3 (23). The common origin with inflammatory cells might explain why osteoclast-induced bone resorption is triggered by proinflammatory cytokines such as IL-1β, TNF-α, OSM, IL-6, IL-11, and IL-17 (16). The mechanism underlying the action of proinflammatory cytokines in bone loss is quite intricate and involves direct mechanisms through binding of cytokines to cytokine receptors expressed by osteoclast precursors, and indirect mechanisms through production of osteoclastogenic factors by inflammatory and resident cells.

Macrophages and osteoclasts share the same progenitor cells, and differentiation of both cells is affected by a loss-of-function mutation in the macrophage-colony stimulating factor (M-CSF) gene (24). The essential role of M-CSF in osteoclastogenesis is also evidenced in mice lacking its receptor c-FMS, encoded by the *Csfr1* gene, which develop severe osteopetrosis (25). The skeletal phenotype caused by deficient M-CSF signaling is due to the essential role of M-CSF on proliferation and survival of osteoclast progenitors (26).

**Figure 1:**
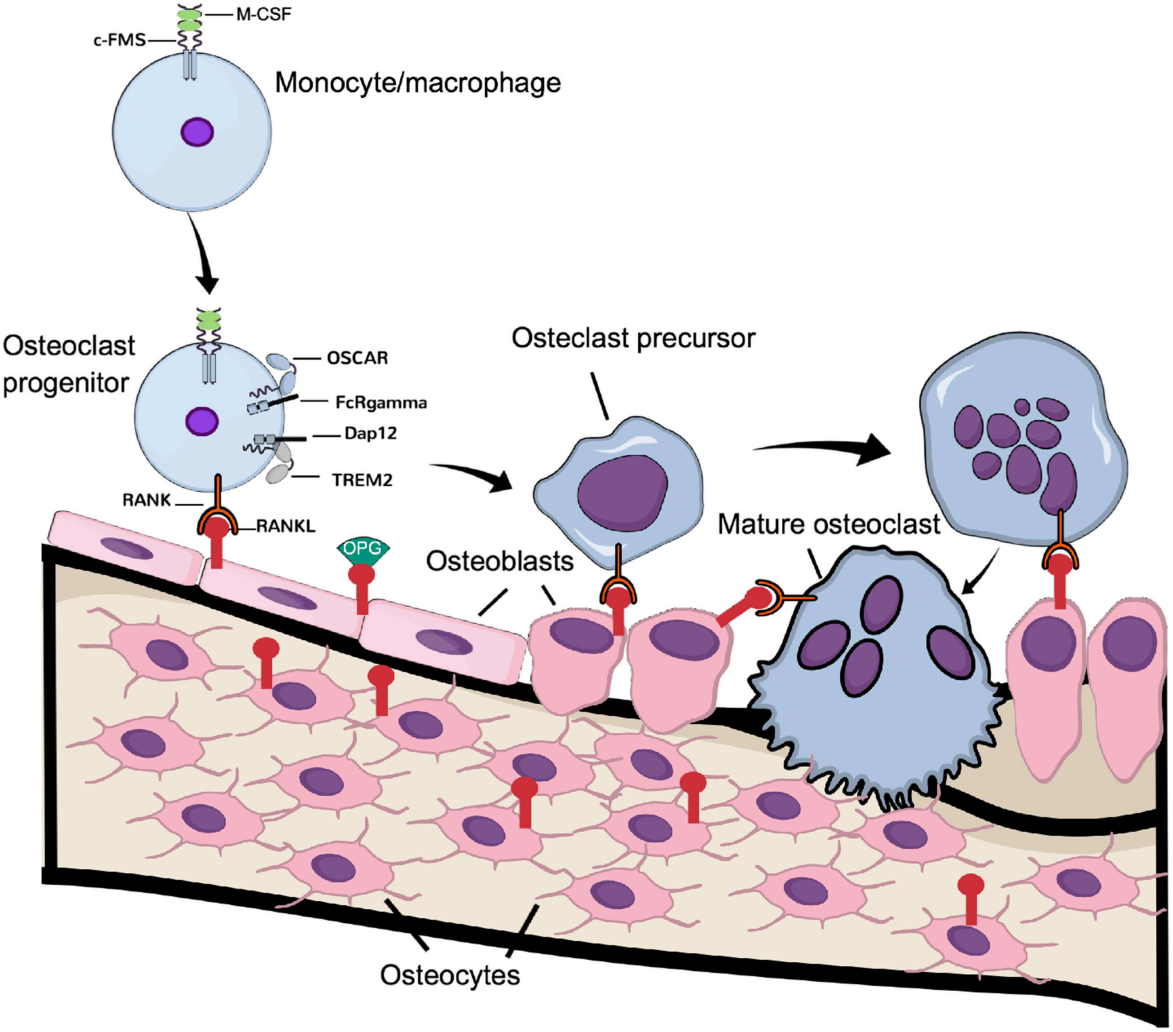
Physiological osteoclast differentiation. Osteoclast progenitors, express c-FMS, the receptor for M-CSF. Upon M-CSF binding to c-Fms, these cells express RANK, which is activated by RANKL expressed by osteoblasts and osteocytes. Binding of RANKL to RANK, in cooperation with the signaling from costimulatory receptors OSCAR/FcRgamma and TREM-2/Dap12, induce differentiation of the progenitor cells to osteoclast precursors, which eventually fuse to latent, multinucleated osteoclasts. Continuous signaling by RANK induces their activation to mature, bone resorbing osteoclasts.

Among the cytokine receptors affecting osteoclastogenesis, a crucial molecule is the receptor RANK (receptor activator of nuclear factor (NF)-κB) (Figure 1). Mice deficient in *Tnfrsf11a* (the gene encoding RANK) have impaired osteoclastogenesis and display severe osteopetrosis (27). RANKL (the ligand for RANK), a cytokine belonging to the tumor-necrosis factor (TNF) superfamily, is expressed by resident bone cells such as osteoblasts and osteocytes (16), and also by different T cells (28), again indicating the active influence of the immune system in osteoclastogenesis. Deletion of the *Tnfsf11* (the gene encoding RANKL) results in mice phenocopying *Tnfrsf11a*^−/−^ mice. Both the formation and activity of mature osteoclasts are stimulated by ligation of RANKL to RANK *in vitro* (29–31). To counteract RANKL action, a decoy receptor lacking a transmembrane domain, osteoprotegerin (OPG), competes with RANK for RANKL binding and blocks osteoclast differentiation and activation (32, 33).

Not only immune cells require costimulatory signals for activation but also osteoclasts require these signals for their activation, in addition to the signaling induced by M-CSF and RANKL (Figure 1). In fact, the immunoreceptor tyrosine-based activation motif (ITAM)-harboring adaptors, Fc receptor common gamma subunit (FcRγ), and DNAX-activating protein (DAP)12 are essential for osteoclast terminal differentiation, as demonstrated in osteopetrotic mice lacking these receptors (34). In osteoclasts, the immunoglobulin-like receptors associated with FcRγ and DAP12 are osteoclast-associated receptor (OSCAR) and the triggering receptor expressed in myeloid cells 2 (TREM-2), respectively (26). Although FcRγ/DAP12 are crucial for osteoclastogenesis to occur, the ligands activating the receptors in osteoclast progenitors are not known. Recently, it was demonstrated that downstream of kinase-3 (DOK3), a protein known to physically interact with DAP12 in macrophages to inhibit TLR signaling (35), is an important negative regulator of osteoclast formation. The mechanism involves inhibition of M-CSF and RANKL-induced activation of Syk and ERK. *In vivo, DOK3*^−/−^ mice have reduced trabecular bone mass and increased number of TRAP^+^ osteoclasts (36).

Since osteoclasts derive from hematopoietic precursors, it is not surprising that TLRs affect osteoclast biology. Being a highly specialized cell, however, activation of TLRs in osteoclasts and their progenitor cells leads to complex outcomes that will be further explored in this review.

## The Toll-Like Receptor Family In Osteoclasts

The TLR family is composed of 13 members in mammals, 10 of which are identified in humans (TLR1-10), among which nine are expressed by osteoclast progenitors (TLR1-9) (37). The members of this family are homologous of the Drosophila Toll protein and consist of integral membrane glycoproteins with extracellular domains of leucine-rich repeats (LRRs), a single transmembrane domain and a C-terminal intracellular domain homologous to the intracellular domain of Interleukin-1 receptor (IL1R), referred to as Toll/IL-1R domain (TIR domain) (38, 39).

**Figure 2:**
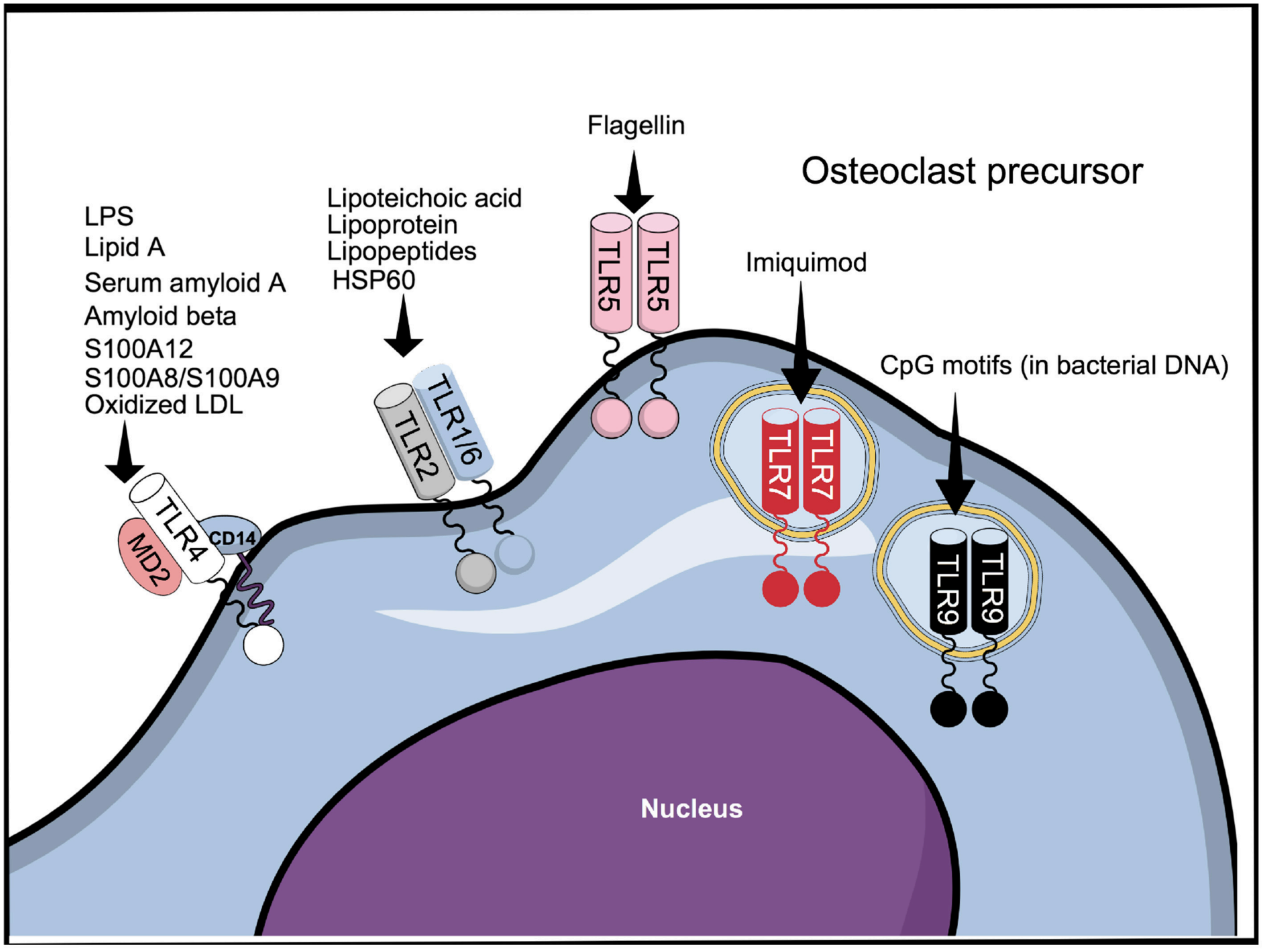
TLR1-9 are expressed by cells belonging to the osteoclast lineage, of which TLR2, 4, 5, 7, and 9 have been shown to be functional. The figure represents all the TLRs that have been described to be expressed in cells belonging to the osteoclast lineage and some of their ligands.

Despite the conserved extracellular LRR domain, TLRs can sense a broad range of MAMPs expressed by invading microbes and danger-associated molecular patterns (DAMPs) expressed by the host, probably by insertions of specific amino acids conferring ligand specificity (40) (Figure 2). Interestingly, different ligands can bind to the same TLR (Figure 2). Thus, TLR4, as an example, can recognize MAMPs such as lipopolysaccharide LPS (41) and lipid A (42), as well as DAMPs such as serum amyloid A (43), S100A8/S100A9 (44), oxidized low-density lipoprotein and amyloid β (45), in addition to several other MAMPs and DAMPs. The capacity to recognize different structures by the TLRs explains why endogenous TLR ligands, such as DAMPs secreted by necrotic cells and extracellular matrix (ECM) in response to tissue damage or injury, as well as MAMPs, such as LPS, lipopeptides, CpG oligodeoxynucleotides, and flagellin, among others, affect osteoclastogenesis. The effects and mechanism of action of MAMPs and DAMPs in osteoclasts is summarized in Table S1 and will be further addressed below.

For signaling, DAMPs and MAMPs associate with TLRs mainly as homo and heterodimers (46). In the case of TLR4, recognition of LPS requires binding to the accessory proteins LPS-binding protein and CD14 before being transferred to the TLR4/MD2 protein complex (47). In addition to TLR4; TLR2, TLR5, and TLR9 are responsible for recognition of bacterial components. TLR2, in association with either TLR1 or TLR6, recognizes various bacterial cell wall components, such as lipoteicoic acid (48) and lipoproteins/lipopeptides (49, 50), while TLR5 mediates the response to flagellin (51) (Figure 2). Similarly to TLR4, and in accordance with their functions, TLR2 and TLR5 are membrane bound. Among the intracellular TLRs, TLR9 recognizes bacterial DNA through CpG motifs (52). The cell response to viruses is manly triggered by the recognition of viral components by the intracellular receptors TLR3, 7, and 8 (53), although it is reported that TLR4 can also recognize viral proteins (54). TLR7 can also be targeted by the synthetic compound imiquimod, used for topical treatment of skin cancers and other cutaneous disorders (55).

Since the cloning of TLR4, it has been shown that TLR4 signals through NF-κB pathway to induce cytokine production (19). Later, several molecules were identified as adapter proteins upstream the activation of NF-κB and other signaling pathways, such as MAPKs, as extensively reviewed elsewhere (56–59).

**Figure 3:**
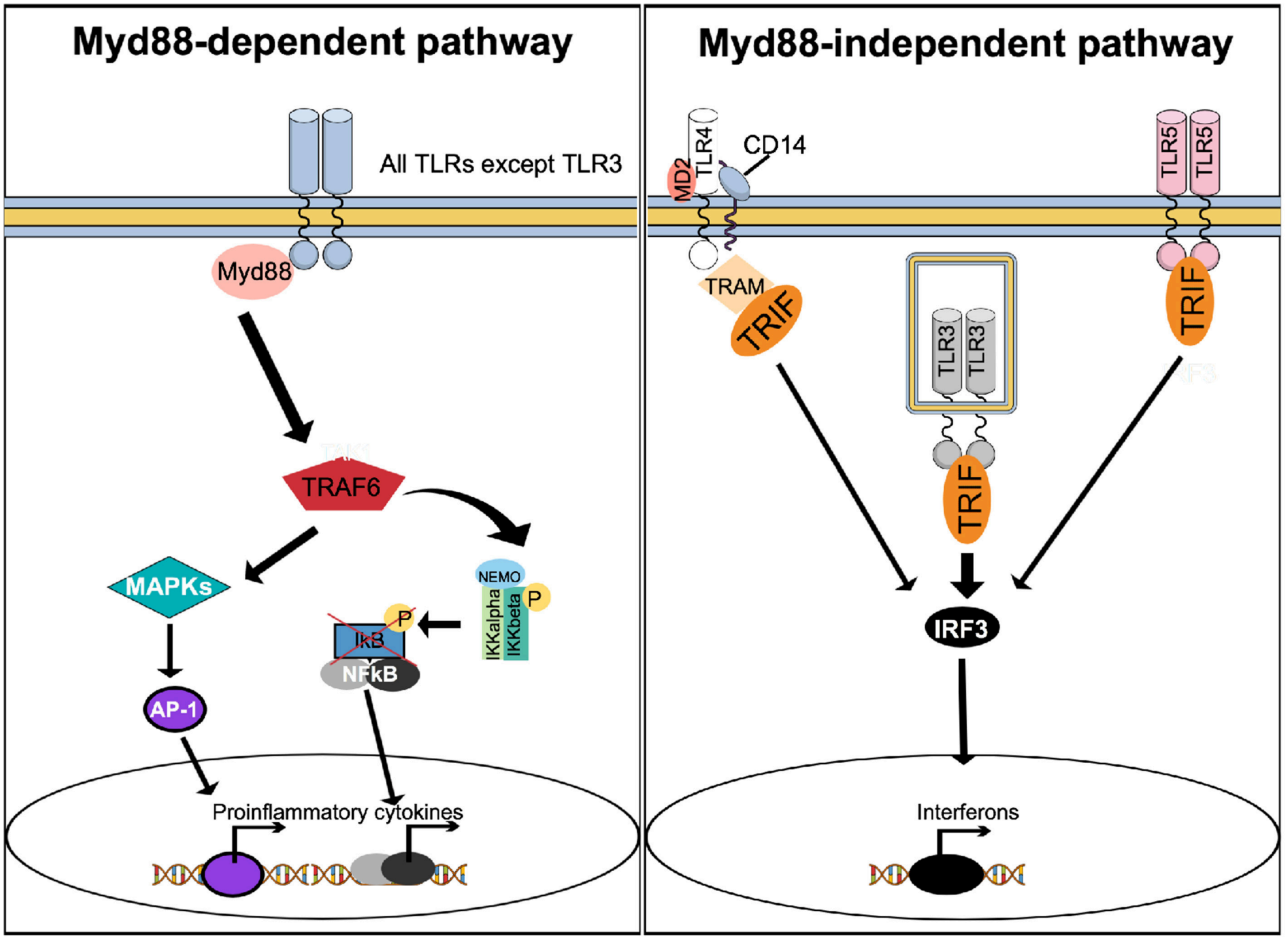
Stimulation of TLRs activates multiple signaling pathways. With exception of TLR3, activation of TLRs results in recruitment of Myd88 to activate the Myd88-dependent canonical pathway. Myd88 activates TRAF6 to form a protein complex capable of phosphorylating the IKK complex, resulting in NF-κB activation. In parallel, the Myd88-dependent pathway results in activation of MAPK and AP-1. The Myd88-dependent pathway results in increased expression of proinflammatory cytokines. The Myd88-independent, non-canonical pathway can be activated by TLR4, TLR3, and TLR5, causing recruitment of TRIF. Unlike TLR3 and TLR5, which recruit TRIF directly to their TIR domain, TLR4 uses TRAM as an adapter protein. TRIF activates IRF3, which translocate to the nucleus to trigger expression of interferon.

To induce effector gene expression, upstream of NF-κB, TLRs use the canonical myeloid differentiation factor 88 (Myd88) pathway and the non-canonical Myd88-independent, TIR-domain-containing adapter-inducing interferon-β (TRIF) pathway (Figure 3). With exception of TLR3, all TLRs activate the Myd88-dependent pathway, while the Myd88-independent pathway can also be activated by TLR3, TLR4, and TLR5 (Figure 3).

Upon agonist binding, a hallmark of TLRs activation is the production of cytokines, including interferons. Activation of the Myd88 pathway leads mainly to the production of pro-inflammatory cytokines, while engagement of TRIF triggers interferon production (60). Since both pro-inflammatory cytokines and interferons are known to affect bone metabolism (16, 61), activation of TLRs can indirectly interfere with osteoclast function.

## TLR Activation In Osteoclasts, Friend or Foe?

Since the pioneering observation showing that LPS from *Bacteroides melaninogenicus* (those days called endotoxin) present in the biofilm in tooth pockets, as well as LPS from *Escherichia coli* and *Salmonella typhii*, could stimulate osteoclast formation, mineral release, and bone matrix degradation in organ cultured fetal rat long bones (14), it has been shown by several groups that LPS from different species of bacteria can stimulate bone resorption *ex vivo* (62–64) and *in vivo* (65–67). Following the discovery of TLRs, it has been found that LPS from several bacteria stimulates osteoclast formation and bone resorption *in vivo* through activation of TLR4 (68, 69), whereas *P. gingivalis* LPS utilizes TLR2 to induce osteoclastogenesis (70, 71). It cannot, however, be determined in these experimental systems if LPS increases osteoclastogenesis by targeting osteoclast progenitor cells, or if osteoclast-supporting cells mediate the effect. The fact that mouse bone marrow macrophages express TLRs (TLR1-TLR9) (72), and that both TLR and RANK recruit TRAF6 to the cytoplasmic tail of the receptors and activate NF-κB, suggests that TLR agonists may, similar to RANKL, stimulate osteoclastogenesis through TLRs present in osteoclast progenitor cells. Using purified bone marrow macrophages/osteoclast progenitors, however, it has been shown that LPS can both inhibit and stimulate osteoclastogenesis dependent on the differentiation level of the progenitors (73). Other studies have demonstrated that LPS can stimulate osteoclast formation also indirectly through enhancing RANKL formation by targeting osteoclast-supporting cells (see further below).

## TLR Activation Inhibits Osteoclastogenesis Stimulated by RANKL

As mentioned above, mouse bone marrow macrophages express TLR1-TLR9, but when these cells are induced to differentiate to mature osteoclasts with RANKL, all receptors, with the exception of TLR2 and TLR4, are downregulated (72). This observation indicates that osteoclast progenitors in bone marrow could be responsive to a variety of TLR agonists. However, despite the fact that the TLR2 agonist *P. gingivalis* activates ERK1/2, p38, JNK, and NF-κB in mouse bone marrow macrophages, similar to RANKL, treatment of the macrophages with M-CSF and *P. gingivalis* does not result in formation of osteoclasts (74). Similar observation has been made by adding either *E. coli* LPS or CpG-ODN to M-CSF-stimulated macrophages to activate TLR4 and TLR9, respectively (75, 76). Interestingly, activation of TLR9 induced the formation of TRAP^+^ mononucleated cells, but no mature osteoclasts were formed. In contrast to RANKL, activation of TLR2 with *P. gingivalis* stimulation did not induce activation of c-Fos or Nfatc1. Given the crucial role of these transcription factors for osteoclast formation, as demonstrated by the lack of osteoclasts and the osteopetrotic skeleton seen in mice with genetic deletion of *Fos* (77) or *Nfatc1* (78), it is apparent that this difference in signaling downstream RANK and TLR2 is the reason why TLR2 activation does not induce osteoclastogenesis. In contrast to these observations, it has recently been reported that the synthetic TLR7 agonist imiquimod stimulated osteoclast formation in M-CSF treated human CD14^+^ monocytes cultured for 21 days, an effect associated with enhanced expression of *Nfatc1* (79).

**Figure 4:**
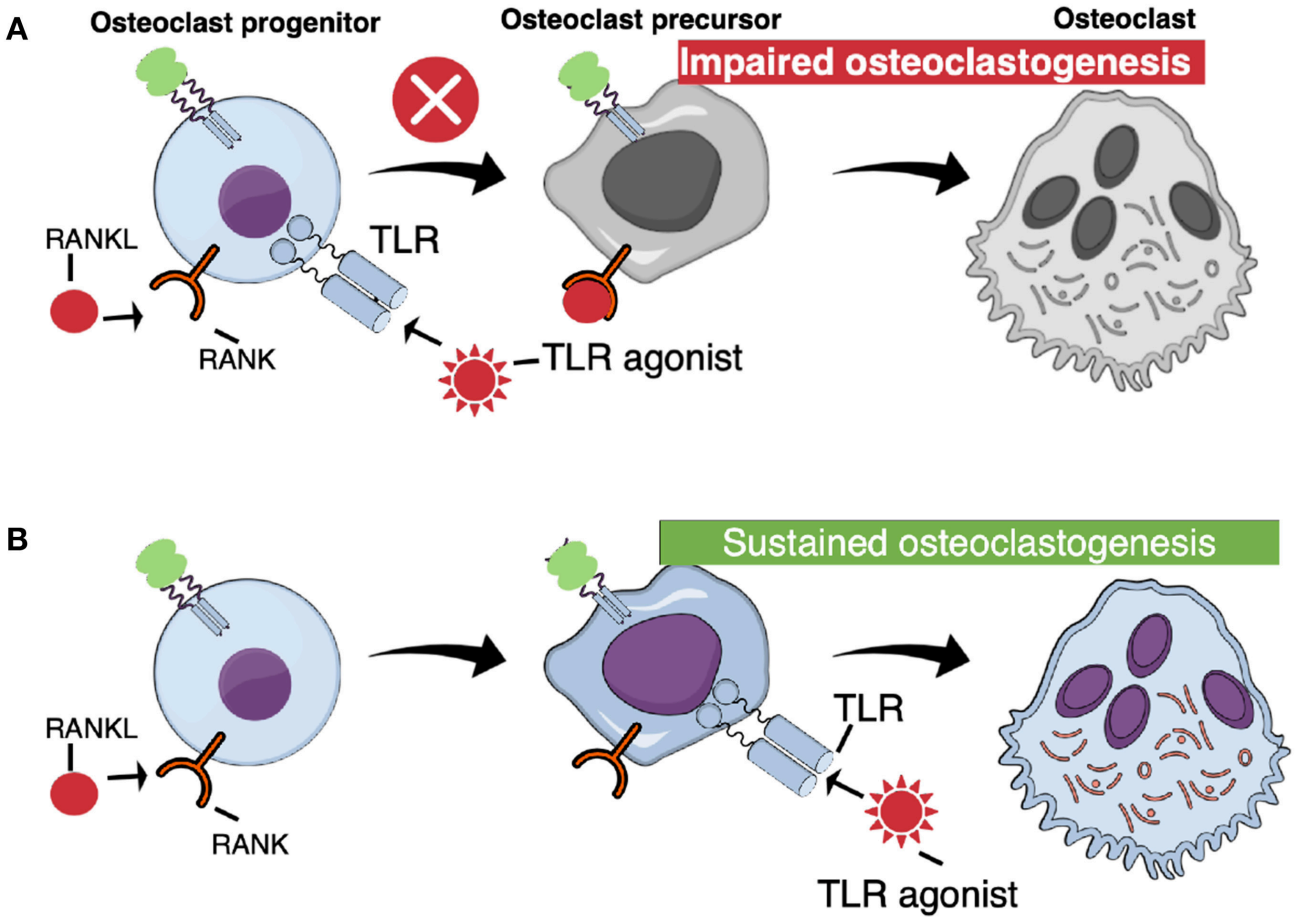
TLR activation at different stages of osteoclast differentiation results in different outcomes. **(A)** When TLR agonists are added at early stages of osteoclast differentiation, concomitant with RANKL, osteoclastogenesis is arrested. **(B)** Committed osteoclast precursors primed with RANKL are capable to differentiate to mature, functional osteoclasts when challenged with TLR agonists in the absence of RANKL.

Surprisingly, activation of TLR in bone marrow macrophages, simultaneously stimulated with RANKL, abolishes osteoclast formation (Figure 4A). Thus, addition of either peptidoglycan from *S. aureus, S. aureus* bacteria, lipoteichoic acid from *S. aureus, P. gingivalis* bacteria, or *P. gingivalis* LPS, which all activate TLR2, or addition of the synthetic TLR2 agonist Pam2CSK_4_ (Pam2), to RANKL-stimulated macrophages, completely blocks osteoclast formation (72, 74, 80–83). Also addition of poly(I:c) dsRNA activating TLR3, *E. coli* LPS activating TLR4, or CpG motif of unmethylated DNA (Cpg-ODN) activating TLR9, blocks RANKL-induced osteoclastogenesis in M-CSF-treated mouse bone marrow macrophage cultures (72, 75, 76, 84). M-CSF/RANKL-stimulated macrophages lose their capacity to phagocyte zymosan, but when co-treated with the TLR agonists, the cells still can phagocyte these particles, demonstrating that they are arrested at the macrophage stage (72). Activation of these four TLRs, also inhibits osteoclast formation in RANKL-stimulated human peripheral blood monocyte cell cultures (72). In agreement with these findings, activation of TLR2 with Pam_3_CSK_4_ (Pam3), or TLR4 with *E. coli* LPS, inhibits osteoclast formation using human CD14^+^ monocytes as progenitor cells, an effect associated with decreased expression of RANK and TREM (84). The TLR2-induced inhibition is dependent on MyD88, but not on TRIF signaling (74). In contrast to activation of TLR2, TLR3, TLR4, and TLR9, activation of TLR5 using flagellin from two different bacteria does not inhibit RANKL-induced osteoclast formation in mouse macrophages expressing TLR5 mRNA and protein (85).

Since osteoclast progenitor cells might be challenged by several agonists activating different TLRs during infectious diseases, the interactions between different TLR agonists have been assessed. Thus, synergistic inhibitory effects on osteoclast formation have been observed when mouse macrophages have been treated with TLR3 together with TLR4, or with TLR4 together with TLR9 (86). These synergistic inhibitions were partially explained by decreased protein expression of the receptor for M-CSF.

### RANKL-Induced Signaling Pathways Are Affected by Activation of TLRs

Similar to RANKL, peptidoglycan from *S. aureus*, poly(I:c) dsRNA, *E. coli* LPS and Cpg-ODN activate NF-κB in mouse macrophages (72), an observation also made in macrophages stimulated with *P. gingivalis* (74). Also similar to RANKL, this bacterium activates ERK1/2, p38 and JNK, both when added alone and when added together with RANKL (74), indicating that inhibition of osteoclastogenesis by TLR2 is not due to decreased phosphorylation of MAPKs. Similarly, *P. gingivalis* did not affect RANKL-induced activation of NF-κB (74). Nor does stimulation of TLR4 with *E. coli* LPS affect RANKL-induced activation of NF-κB, ERK1/2 or p38 (76). Importantly, however, activation of TLR2 with *P. gingivalis*, or TLR4 with *E. coli* LPS, inhibits RANKL-induced activation of Nfatc1, which explains why these TLRs block osteoclastogenesis (74, 76). Activation of TLR2 also inhibited c-Fos induction by RANKL, which is an additional mechanism by which osteoclast formation is decreased. Since c-Fos is a transcription factor upstream of Nfatc1 (87), it is likely that regulation of c-Fos is the reason why Nfatc1 is decreased. Also activation of TLR9 inhibits RANKL-induced c-Fos, by a mechanism due to increased degradation of both c-Fos mRNA and protein (88). This might be due to that the activation of ERK1/2 by CpG-ODN is transient, whereas RANKL causes a sustained activation of ERK1/2, a difference which is explained by the finding that CpG-ODN, but not RANKL, induces the expression of the phosphatase PP2A (88).

Serum amyloid A is a circulating, danger-associated, liver protein which is upregulated by inflammatory processes and which binds to TLR2 (89). This protein also inhibits RANKL-stimulated osteoclast formation in mouse bone marrow macrophage (BMM) cultures (90). The inhibition is associated with decreased expression of RANKL-induced *Fos* and *Nfatc1* mRNA expression, increased expression of the macrophage transcription factors *Mafb* and *Irf8*, as well as with decreased expression of c-Fms protein on the surface of the progenitor cells due to enhanced ectodomain shedding.

### Cytokines Involved in TLR-Induced Inhibition of Osteoclastogenesis

In agreement with the fact that increased formation of inflammatory cytokines is a well-known, Myd88-dependent, phenomenon in macrophages stimulated by TLR agonist, it has been observed that activation of BMMs with peptidoglycan, poly(I:c)dsRNA, *E. coli* LPS, CpG-ODN results in increased expression of TNF-α (72, 75). The expression of *Tnfsf2* (encoding TNF-α), as well as the mRNA expression of *Il6*, and *Il12p40*, is upregulated after stimulation with *P. gingivalis*, whereas RANKL does not affect the expression of any of these cytokines (74). The expression of *Il12p40* mRNA and IL-12 protein is increased also by CpG-ODN (91). Since neutralization of IL-12 partially rescued the inhibitory effect by CpG-ODN on osteoclast formation, and since IL-12 is an inhibitor of osteoclast differentiation (92), it seems induction of anti-osteoclastogenic cytokines by TLR9 might partially explain the inhibitory effect on osteoclastogenesis.

Not only inflammatory cytokines are induced by TLR signaling, but also type I interferons are induced through the TRIF-mediated pathway (Figure 3). Since IFN-β is a negative feedback regulator of RANKL-induced osteoclast formation due to decreased expression of c-Fos protein (93), the possibility exists that IFN-β may be important for decreased osteoclast formation caused by activation of TLR2 and TLR4. The observations showing that TLR2- and TLR4-induced inhibition of *RANK* expression and human osteoclast formation is independent of IFN-β (84) and that TLR2-induced inhibition of human osteoclastogenesis is dependent on Myd88, but not TRIF, argues for that IFN-β is not involved in the decreased osteoclast formation caused by activation of TLR2 or TLR4. Most recently, however, it has been reported that haptoglobin decreased osteoclast formation *in vivo* and *in vitro* through activation of TLR4 and induction of IFN-β (94). Thus, haptoglobin deficient mice have low trabecular bone mass and increased numbers of osteoclasts, with no effect on osteoblast numbers. Treatment of mice locally with haptoglobin results in decreased osteoclast formation in mice co-stimulated by RANKL injections. In mouse BMM cultures, haptoglobin decreases osteoclast formation by a mechanism dependent on TLR4, but not on TLR2 or TLR7, and associated with increased mRNA and protein expression of IFN-β. The inhibitory effect was abolished by antibodies neutralizing IFN-β. Similar to previous findings (93) increased IFN-β and decreased osteoclast formation was associated with unaffected mRNA expression of *Fos* but decreased c-Fos protein expression. It was, however, surprising that haptoglobin did not induce phosphorylation of IRF-3, which is a well-known inducer of IFN-β in the TRIF pathway activated by TLRs (Figure 3). It, therefore, remains to be understood why TLRs and haptoglobin induce IFN-β by seemingly different mechanisms in osteoclast progenitor cells. It also remains to be understood why TLR-induced inhibition of osteoclast differentiation in human osteoclast progenitors is independent of IFN-β, whereas activation of TLR4 by haptoglobin in mouse osteoclast progenitors is dependent.

IL-1 receptors, similar to TLRs, have a cytosolic TIR domain, and also share several common downstream signaling pathways. It has, therefore, been investigated how activation of IL-1 receptors affect RANKL-induced osteoclast formation. Lee et al., using human CD14^+^ monocytes, found that IL-1β also inhibited RANKL-stimulated osteoclast formation, when the cells were co-stimulated with the two cytokines (95). In contrast, Chen et al., using mouse bone marrow macrophages, found that IL-1α, in contrast to *P. gingivalis* LPS, enhanced osteoclast formation induced by RANKL (81). IL-1α-induced stimulation was observed with both stimulatory and permissive concentrations of RANKL. Both the inhibitory effect by *P. gingivalis* LPS and the stimulatory by IL-1α were dependent on Myd88. The diverse responses were explained by the observation that LPS abrogated the RANKL-induced expression of *Blimp1*, a transcriptional repressor of the anti-osteoclastogenic transcription factors IRF8 and MafB, whereas IL-1α potentiated RANKL-induced expression of *Blimp1*.

### Comparison of Effects by TLRs on Osteoclast Formation *in vitro* and *in vivo*

The inhibitory effects by activation of TLRs on osteoclast formation does not explain why infections with *E. coli, S. aureus*, or *P. gingivalis* result in increased formation of osteoclasts and bone resorption (96). It has been suggested, however, that the inhibition of osteoclast formation by TLR may be part of a homeostatic mechanism limiting bone resorption during infection and inflammation (84). It might also be possible that the inhibitory effect is a mechanism to increase the number of macrophages involved in the defense against the bacterial infections.

The inhibition of osteoclastogenesis by TLR agonists seems to be specific to un-committed purified mouse bone marrow macrophages and human peripheral blood monocytes, since *P. gingivalis* LPS, *S. aureus* and Pam2 do not inhibit bone resorption in RANKL-stimulated mouse calvarial bones *ex vivo* (82, 83). Nor do these agonists inhibit osteoclast formation in RANKL-stimulated calvarial periosteal cell cultures containing osteoclast progenitors. This may be of particular interest since formation of mature osteoclasts only takes place on bone surfaces, not in bone marrow. The reason why the osteoclast progenitors in the periosteum is not inhibited by TLR agonists is not known, but may be due that these cells do not express TLRs, or that these cells are committed osteoclast progenitors, or that surrounding non-osteoclastic cells make the osteoclast progenitors insensitive to TLR-induced inhibition.

## TLR Activation Induces Osteoclastogenesis in RANKL-Primed Cells

In contrast to the inhibition of un-committed osteoclast progenitors in bone marrow or peripheral blood, activation of TLR in RANKL-committed osteoclast progenitors from bone marrow results in stimulation of osteoclastogenesis (Figure 4B). Zou et al. were the first to show that mouse bone marrow macrophages primed with M-CSF/RANKL, and then treated with *E. coli* LPS and M-CSF, in the absence of RANKL, differentiate to mature osteoclasts (97). Under these conditions, LPS induced the expression of IL-1β and TNF-α, and addition of antibodies neutralizing TNF-α inhibited osteoclast stimulation by LPS, in agreement with previous studies showing that the stimulatory effect of LPS *in vivo* on the numbers of osteoclast progenitors in bone marrow is inhibited in mice deficient of the *p55* TNF receptor (67). In contrast, inhibition of IL-1β with the IL-1 receptor antagonist did not affect LPS-induced stimulation of osteoclast formation in RANKL-primed cells. The effect of commitment by RANKL is long-lasting and *E. coli* LPS is able to induce osteoclastogenesis several days after priming (76). Under these conditions, LPS does not decrease the expression of Nfatc1, in contrast to the inhibition seen when LPS is added together with RANKL to non-committed cells. Also addition of *P. gingivalis* to RANKL-primed cells results in osteoclast formation (74). Similar induction of osteoclast formation is obtained by adding other TLR2 agonists, such as formaldehyde-inactivated *S. aureus*, Pam2 and Pam3 (83, 98). At variance, Kassem et al. found that UV-light inactivated *S. aureus, P. gingivalis* LPS and heat-killed *Listeria monocytogenes* cause increased numbers of TRAP^+^ mononucleated cells in RANKL-primed bone marrow macrophage cultures. These cells expressed enhanced mRNA levels of *Acp5* (encoding TRAP), *Ctsk* (encoding cathepsin K), *c-Fos*, and *Nfatc1*, but did not form multinucleated osteoclasts. In contrast, Pam2 and Pam3 robustly stimulated formation of multinucleated osteoclasts. Activation of TLR9 with CpG-ODN in RANKL-primed cells also results in formation of multinucleated osteoclasts and, similar to activation of TLR4, activation by CpG-ODN is dependent on TNF-α (75). Synergistic stimulation of osteoclastogenesis in RANKL-primed cells by co-treatment with either TLR3/TLR9 agonists, or TLR4/TLR9 agonists, has also been observed (86).

Since TLR2 and TLR4 are not downregulated during osteoclastogenesis (72), the role of these receptors in mature osteoclasts has been assessed. Three studies have demonstrated that activation of TLR2 with peptidoglycan from *S. aureus*, or of TLR4 with *E. coli* LPS, increases the survival of mature osteoclasts (72, 76, 99), an observation not seen by adding agonists activating TLR3 or TLR9.

It is apparent that TLRs have dual effects on osteoclastogenesis dependent on the differentiation status of osteoclasts or their progenitors. The exact molecular mechanisms causing osteoclast progenitors to respond to TLR agonists with enhanced differentiation along the osteoclastic lineage, provided the cells are primed with RANKL, and then exposed to TLR agonists in the absence of RANKL remains to be shown. Another important issue is if the dual actions also are occurring *in vivo*. It is well-documented in several experimental systems that LPS induces osteoclast formation and bone loss *in vivo*, which means that the overall effect is that of a stimulation of osteoclastogenesis.

### Indirect Activation of Osteoclastogenesis by TLRs

**Figure 5:**
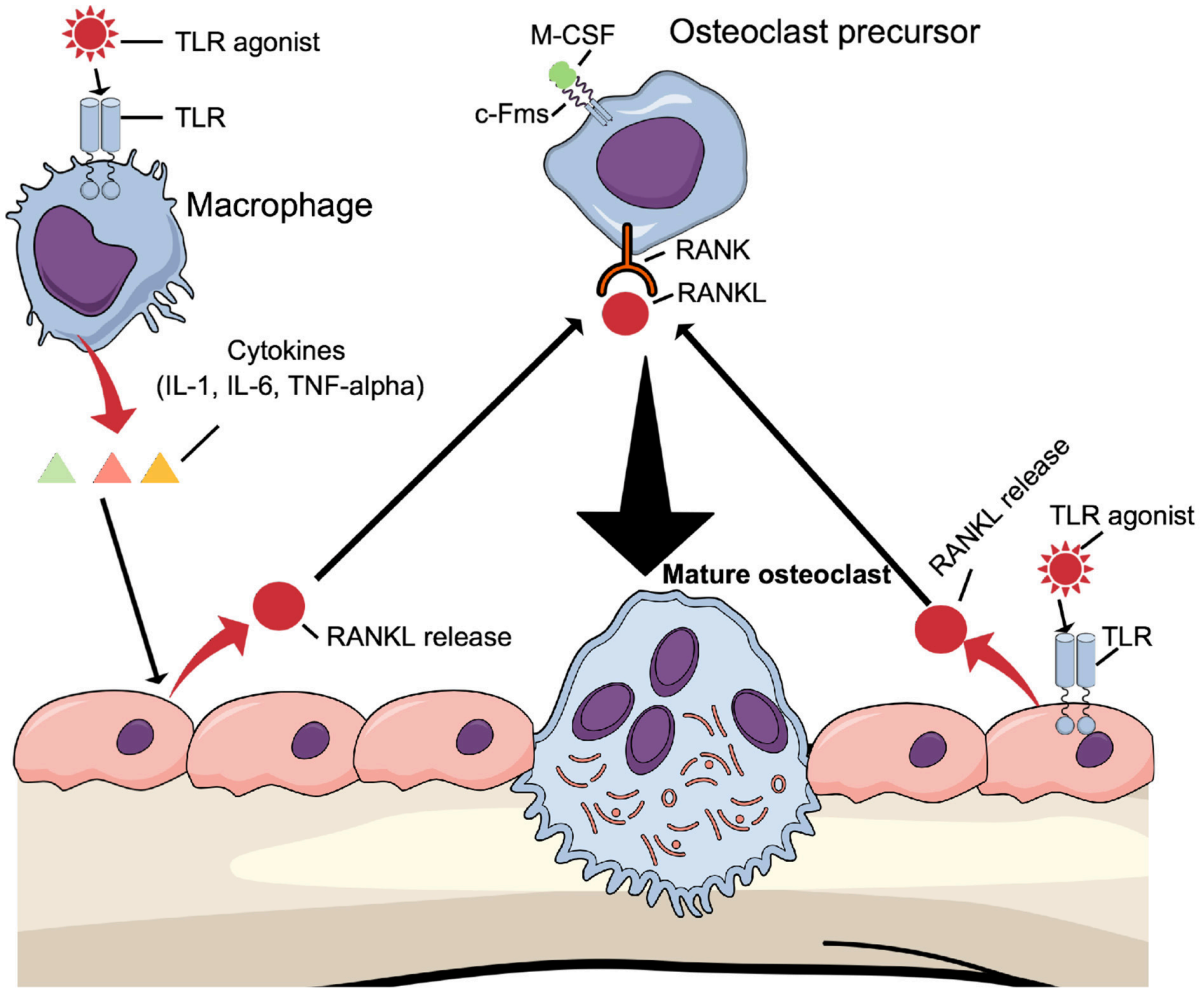
Osteoclastogenesis can be induced indirectly by TLR agonists TLR agonists induce the expression of proinflammatory, pro-osteoclastogenic cytokines such as IL-1β, IL-6, and TNF-α by macrophages, which will bind to cytokine receptors expressed in osteoblasts causing induction of RANKL expression. Alternatively, TLR agonists bind to TLRs expressed by osteoblasts to induce RANKL expression. In both cases, RANKL will induce differentiation of osteoclast precursors to mature osteoclasts.

One mechanism by which TLR activation induces osteoclast formation *in vivo* may be through the above-described mechanism, where TLR agonists directly enhance osteoclastogenesis in committed osteoclasts. Another mechanism may be due to increased expression of osteoclast-stimulating cytokines (16). These cytokines induce osteoclast formation indirectly by increasing the expression of production of RANKL in osteoblasts/osteocytes (Figure 5, left part). The possibility also exists that TLR agonists enhance osteoclast differentiation indirectly by regulating the production of RANKL and OPG in osteoblasts (Figure 5, right part). The fact that osteoblasts express TLR2, TLR4, TLR5, TLR6, and TLR9 further support such a possibility (82, 85, 100, 101).

Stimulation of TLR4 with LPS from either *E. coli* or *Actinobacillus actinomycetemcomitans* increases the mRNA expression of *Tnfsf11* in mouse calvarial osteoblasts, the osteoblastic cell line MC-3T3E1 and the stromal cell line ST-2 (100). This effect was independent of TNF-α. In contrast to activation of TLR4, activation of TLR9 with CpG-ODN does not induce *Tnfsf11* mRNA in osteoblasts, although both *E. coli* LPS and CpG-ODN stimulated the expression of TNF-α and activated NF-κB, ERK1/2 and p38 (101). Using co-cultures of osteoblasts and bone marrow macrophages from wild type mice and mice deficient in either *Tlr4* or *Tlr9*, it has been shown that both LPS and CpG-ODN stimulate osteoclast differentiation, but that the effect of CpG-ODN is more dependent on TLR9 receptors in macrophages than those in osteoblasts (102). In contrast, the effect of LPS was dependent on TLR4 in osteoblasts.

Activation of TLR2 in mouse calvarial osteoblasts by a variety of agonists (*P. gingivalis* LPS, *S. aureus*, Pam2, Pam3, heat-killed *Listeria monocytogenes*, and lipoprotein from *Mycoplasma salivarium*) increases *Tnfsf11* mRNA expression, depending on Myd88, but independent of IL-1β, IL-6 or TNF-α, without affecting the mRNA expression of *Tnfrsf11b* (encoding OPG) (82, 83). The agonists activated NF-κB and the effect on *Tnfsf11* expression could be inhibited by Celastrol, an inhibitor of IκB kinase. A similar stimulation of *Tnfsf11* mRNA and RANKL protein, with no effect on *Tnfrsf11b* mRNA and OPG protein, was observed in mouse calvarial bones *ex vivo* stimulated by *P. gingivalis* LPS, *S. aureus* and Pam2, which resulted in increased osteoclast formation and bone resorption in the calvarial bones, independent of the IL-1β, IL-6 and TNF-α (82, 83). Treatment of mice *in vivo* with *P. gingivalis* LPS or Pam2 also resulted in increased mRNA expression of *Tnfsf11*, no effect on *Tnfrsf11b* mRNA, enhanced osteoclast formation and bone loss, effects which were absent in *Tlr2*^−/−^ mice (82). Increased mRNA expression of *Tnfsf11* and soluble RANKL protein has also been observed in synovial fibroblasts from patients with rheumatoid arthritis (79).

Further support for a TLR-dependent indirect mechanism stimulating osteoclast formation comes from experiments showing that LPS, stimulating TLR4, and diacyl lipopeptide, stimulating TLR2, enhances osteoclast formation in cocultures of mouse osteoblasts and bone marrow macrophages (103). The effect on both LPS and diacyl lipopeptide, but not osteoclast formation induced by 1,25(OH)_2_-vitamin D3, was dependent on Myd88, but not TRIF, and associated with increased mRNA expression of *Tnfsf11*, which most likely was the reason for the stimulatory effect on osteoclast formation although not formally shown.

Activation of TLR5 in mouse calvarial osteoblasts with flagellin from two different bacteria also results in increased mRNA expression of *Tnfsf11*, but, in contrast to activation of TLR2, flagellin decreases *Tnfrsf11b* mRNA in the osteoblasts (85). Stimulation of *Tnfsf11* mRNA by flagellin was dependent on Myd88, but independent on IL-1β, IL-6, and TNF-α. Similar to activation of TLR2, flagellin activated NF-κB and stimulation of *Tnfsf11* mRNA was inhibited by two different IκB kinase inhibitors. Increased *Tnfsf11* mRNA and RANKL protein, and decreased *Tnfrsf11b* mRNA and OPG protein, was also observed in mouse calvarial bones *ex vivo*, and treatment with flagellin increased osteoclast formation and bone resorption in the calvaria. A similar increase of *Tnfsf11* mRNA and decrease of *Opg* mRNA can be seen in mice treated with flagellin, causing increased osteoclast formation and extensive bone loss in wild type, but not in *Tlr5*^−/−^ mice (85).

Another indirect mechanism by which TLRs can stimulate osteoclastogenesis is through TLR2-induced upregulation of the chemokine CXCL10 (104). Stimulation of mouse calvarial osteoblasts with Pam3 results in increased mRNA expression of *Cxcl10* and CXCL10 protein. When supernatants from Pam3-stimulated osteoblasts were added to RANKL-stimulated cultures of the RAW264.7 cell line it was observed that the supernatants potentiated the osteoclastogenic effect of RANKL by a mechanism that could be inhibited by antibodies neutralizing CXCL10.

## Conclusion

Altogether, observations on osteoclast progenitors and osteoblasts, as well as findings in organ cultures and *in vivo*, demonstrate that TLRs can increase osteoclast formation and bone resorption by several mechanisms. In cell cultures, TLRs also can arrest osteoclast differentiation when acting on un-committed progenitors cells by interfering with RANKL-induced signaling. The importance of TLRs in osteoblasts and osteoclast progenitors *in vivo* must await studies using mice with cell specific deletions of different TLRs in these bone cells.

## Author Contributions

PS and UL wrote the manuscript and approved it for publication.

### Conflict of Interest Statement

The authors declare that the research was conducted in the absence of any commercial or financial relationships that could be construed as a potential conflict of interest.
