# Supplementary material for: Finding a Toll on the Route: The Fate of Osteoclast Progenitors After Toll-Like Receptor Activation
Source: Front Immunol. 2019 Jul 17;10:1663. doi: 10.3389/fimmu.2019.01663 (PMC6652233; doi:10.3389/fimmu.2019.01663)
Supplement: Supplementary file 1 [file Table_1.DOCX]

Table 1 – Summary of the main *in vitro* findings related to mechanisms involved in modulation of osteoclastogenesis by TLR ligands.

| Source | Product | Receptor | Experimental System | Outcome | Mechanism | Reference |
| --- | --- | --- | --- | --- | --- | --- |
| *Dental plaque* | Whole dental plaque deposit | ? | Bone organ culture (radius or ulna from 19-day old rat fetuses) | Supernatants of leucocytes stimulated with dental plaque stimulate bone resorption. | ? | (Horton, Raisz et al. 1972) |
| *Bacterioides melaninogenicus* | Endotoxin/LPS | ? | Fetal bone organ culture | Endotoxin caused bone resorption assessed by the release of previously incorporated ^45^Ca in the bone matrix. | ? | (Hausmann, Raisz et al. 1970) |
| *Actinobacillus actinomycetemcomitans* | LPS | ? | Mouse calvaria organ culture | LPS-induced calcium release is partially inhibited by indomethacin.  LPS-induced calcium release is partially inhibited by anti-IL-1 serum. | PGE2 and IL-1 participate on LPS-induced bone resorption *in vitro*. | (Ishihara, Nishihara et al. 1991) |
| Endogenous | Serum amyloid A | ? | Mouse BMMs | Serum amyloid A inhibits RANKL-induced osteoclastogenesis. | Serum amyloid A inhibits RANKL-induced upregulation of osteoclast genes (eg. OSCAR, TRAP, c-Fos and NFATc1) and reverses the reduction of MafB and IRF8 by RANKL. Serum amyloid A decreases c-Fms expression via ectodomain shedding. | (Oh, Lee et al. 2015) |
| *Porphyromonas gingivalis* | LPS | TLR2 | Primary uncommitted BMMs | Simultaneous treatment of BMMs with LPS-PG and RANKL abrogates RANKL-induced osteoclast formation | While RANKL alone induces NFATc1 expression, simultaneous treatment of BMMs with RANKL and LPS impairs NFATc1-induced RANKL expression.  LPS from *P. gingivalis* blocks RANKL-induced expression of Blimp1, a transcriptional repressor of anti-osteoclastogenic genes. | (Chen, Su et al. 2015) |
| *Staphylococcus aureus* | Whole bacteria inactivated by UV-light | TLR2 | Mouse calvarial organ cultures, isolated parietal cells and BMMs. | *S. aureus* stimulates bone resorption in parietal bones and bone formation in parietal cells, but abolishes RANKL-stimulated osteoclastogeneis in BMMs. | *S. aureus* binds to TLR2 to induce RANKL in osteoblasts. | (Kassem, Lindholm et al. 2016) |
| Synthetic | Pam_3_CSK_4_ | TLR2 | Human and mouse osteoclast precursors (RANKL-primed) | Treatment of the precursors with Pam_3_CSK_4_ inhibits osteoclastogenesis when added before or together with RANKL. | Inhibition of RANK and TREM2 expression. | (Ji, Park-Min et al. 2009) |
| Endogenous | Serum amyloid A | TLR2 | BMMs, calvarial osteoblasts and cocultures of these two cell types | Serum amyloid A inhibits RANKL-induced osteoclastogenesis, but slightly increases osteoclastogenesis in the coculture system. | Serum amyloid A binds to TLR2 in osteoclast precursors to interfere with IRF8 downregulation and c-fos and NFATc1 activation. | (Kim, Yang et al. 2016) |
| *Staphylococcus aureus* | Lipoteichoic acid (LTA) | TLR2 | Mouse BMMs | LTA inhibit osteoclastogenesis when added together with RANKL. This effect is absent in TLR2 knockout cells, but just partially inhibited in Myd88 knockout cells. LTA prevents AP-1 binding stimulated by MCSF and RANKL treatment, but NFATc1 binding. | LTA inhibition of osteoclastogenesis is due to activation of TLR2, but just partially dependent on Myd88. AP1, but not NFATc1 binding, is affected by LTA treatment during osteoclast differentiation. | (Yang, Ryu et al. 2009) |
| *Porphyromonas gingivalis* | LPS | TLR2 | BMMs | LPS is unable to induce osteoclastogeneis in the presence of MCSF. LPS inhibit RANKL-induced osteoclastogenesis from non-commited osteoclast precursors, but potentiates osteoclastogenesis of commited precursors. | LPS inhibit osteoclastogenesis through TLR2-Myd88, but not TLR4-TRIF. RANKL suppresses LPS-induced cytokine production down-regulating TLR/NF-κB and up-regulating NFATc1 | (Zhang, Liu et al. 2011) |
| *Staphylococus aureus, Synthetic* | Whole bacteria, Pam_2_CSK_4,_ Pam_3_CSK_4_ | TLR2 | Committed osteoclast precursors and *in vivo* bone resorption induced by intraperitoneal injection in mice | Wild type *S. aureus*, but not the lipoprotein-deficient bacteria, induces bone resorption *in vivo*. Pam_2_CSK_4_ and Pam_3_CSK_4_ induce bone resorption and osteoclast differentiation *in vivo* and osteoclastogenesis from committed precursors *in vitro*. | Lipoproteins induce osteoclastogenesis by direct and indirect mechanisms. The direct mechanisms involve activation of TLR2-Myd88 axis, MAPKs and NF-κB. The indirect mechanisms involve TNF-α and IL-6 production by osteoclast precursors and RANKL production by osteoblasts. | (Kim, Yang et al. 2013) |
| *Porphyromonas gingivalis/*Synthetic | LPS, Heat-killed *L. Monocytogenes* (HKLM), FSL-1 Pam_2_CSK_4,_ Pam_3_CSK_4_ | TLR2 | *In vivo* injection in mice, mouse organ-cultured calvarial bones, isolated murine osteoblasts, BMMs | LPS, HKLM, FSL1, Pam_2_CSK_4_ and Pam_3_CSK_4_stimulate *Tnfsf11* expression, bone resorption and osteoclast formation in calvarial bones *in vivo* and *ex vivo.* The effect of all agonists was absent in bones from TLR2^-/-^ mice. LPS and Pam_2_CSK_4_ inhibit RANKL-induced osteoclastogenesis in osteoclast precursors. | *P. gingivalis* LPS binds to TLR2 to activate Myd88 and NF- κB and stimulate RANKL. | (Kassem, Henning et al. 2015) |
| Endogenous | C5a/Pam_3_CSK_4_ | C5aR/TLR2 | Mouse osteoblasts | Osteoblasts treated with C5a and/or Pam_3_CSK_4_ increase the production of CXCL10. Supernatants of these osteoblasts induce osteoclastogenesis in RAW 264.7 macrophages.. | C5a-stimulation of osteoblasts leads to activation of signaling pathways and expression of genes that are in common with TLR activation. C5aR1 is expressed in osteoblasts and coimmunoprecipitates with TLR2. C5a treatment in osteoblasts triggers the activation of multiple signaling pathways and leads to the generation of CXCL10, a pro-osteoclastogenic factor. The effect of C5a is potentiated by Pam_3_CSK_4_ | (Modinger, Rapp et al. 2018) |
| *Echerichia coli, Synthetic* | LPS/Diacyl lipopeptide (FSL-1) | TLR2 and TLR4 | Coculture of osteoblasts and hematopoietic cells. Mature osteoclasts. BMMs. | LPS and FLS-1 induce osteoclastogenesis in the coculture system, effect that is not observed in Myd88^-/-^ mice. LPS, but not FSL-1, supports survivals of mature osteoclasts. Myd88^-/-^ mice exhibit osteopenia. | Activation of Myd88 and downstream signaling through PKC and MEK/ERK is important for RANKL production by osteoblasts. Mature osteoclasts lack TLR6, making them insensitive to FSL-1. Myd88 is important for bone cells response to TLR agonists. | (Sato, Takahashi et al. 2004) |
| *Escherichia coli* | LPS | TLR4 | Mouse BMMs | LPS inhibits RANKL-induced osteoclastogenesis when added together with RANKL, but stimulates osteoclastogenesis in RANKL-primed cells, even in the absence of RANKL. | The effect of LPS inducing osteoclastogenesis in RANKL primed cells is partially blocked by anti-TNF-α and anti TNFR1 antibodies, indicating the participation of TNF-α in LPS effects. | (Zou and Bar-Shavit 2002) |
| *Escherichia coli* | LPS | TLR4 | Mouse embryonic calvarial cells | IFN-γ inhibit CD-14 and IL1β expression and consequently suppresses osteoclastogenesis | IFN-γ suppresses LPS stimulated osteoclastogenesis and by inhibiting CD-14 and IL-1β up-regulation | (Amano, Kawakami et al. 1997) |
| *Escherichia coli* | LPS | TLR4 | BMMs | LPS added in combination with MCSF stimulates osteoclastogenesis from bone marrow cells isolated from Src homology 2-domain phosphatase-1(SHP-1)-defective mice. | Mice lacking SHP-1 acquire a novel pathway for LPS-induced osteoclastogenesis.. | (Hayashi, Tsuneto et al. 2004) |
| *Escherichia coli* | LPS | TLR4 | Mouse BMMs, calvarial osteoblasts and cocultures of these two cell types | LPS inhibit osteoclastogenesis of uncommitted BMMs, but stimulates osteoclastogenesis in cocultures of BMMs and osteoblasts and in RANKL-primed BMMs. | LPS inhibits RANKL-induced JKN activation and NFATc1 expression in uncommitted BMMs. LPS activates Akt, NF-κB and ERK pathways to promote osteoclast survival. | (Liu, Wang et al. 2009) |
| *Escherichia coli* | LPS | TLR4 | Human and mouse osteoclast precursors | Treatment of the precursors with LPS inhibits osteoclastogenesis when added before or together with RANKL. | Inhibition of RANK and TREM2 expression. | (Ji, Park-Min et al. 2009) |
| *Escherichia coli* | LPS | TLR4 | LPS injection and ex vivo culture of bone marrow cells in the presence of 1,25(OH)_2_D3 and dexamethasone | LPS induces osteoclastogenesis and increases the number of osteoclast precursors when injected in wild type mice, but not in p55TNF receptor knockout mice | LPS induced osteoclastogenesis is mediated by Type I TNF receptor. | (Abu-Amer, Ross et al. 1997) |
| *Escherichia coli* | LPS | TLR4 | Mouse embryonic calvarial cells | LPS-induced osteoclastogenesis is inhibited by blocking of CD14 | LPS-induced osteoclastogenesis is dependente on CD14 | (Amano, Kawakami et al. 1997) |
| *Escherichia coli* | LPS | TLR4 | Mouse embryonic calvarial cells | LPS-induced osteoclastogenesis is inhibited by blocking of IL-1β | LPS-induced osteoclastogenesis is dependent on IL-1β | (Amano, Kawakami et al. 1997) |
| *Escherichia coli* | LPS | TLR4 | Mature osteoclasts | LPS promotes osteoclast survival and this phenomenon is not inhibited by OPG or IL-1Ra. LPS stimulates osteoclast survival of osteoclasts derived from TNFRI knockout mice. | LPS promotes osteoclast survival through TLR4, but independently of IL-1, RANKL and TNF-α. | (Itoh, Udagawa et al. 2003) |
| *Salmonella typhimurium* | Flagellin | TLR5 | *In vivo* injection in mice, mouse organ-cultured calvarial bones, isolated murine osteoblasts, BMMs | Flagellin stimulates bone resorption *in vivo* and in organ culture.  Flagellin does not inhibit RANKL-induced osteoclastogenesis from osteoclast precursors *in vitro*. | Flagellin, through TLR5, up-regulates RANKL, dependent on Myd88 and NF-κB. | (Kassem, Henning et al. 2015) |
| Synthetic | Imoquimod | TLR7 | Synovial fibroblasts, peripheral blood CD14^+^ human monocytes and their cocultures. | Imoquimod directly induces differentiation of monocytes when in combination with MCSF, in the absence of RANKL. In cocultures of reumathoid arthritis synovial fibroblasts and monocytes, pretreatment of fibroblasts with imoquimod potentiated osteoclastogenesis. | Inhibition of p38  MAPK, ERK, NF-k B, NFATc1, AP-1 and JAK2, decreases osteoclastogenesis induced by imoquimod, suggesting the participation of these pathways. | (Kim, Kim et al. 2019) |
| Synthetic | CpG-oligodeoxynucleotides (CpG-ODN) | TLR9 | Mouse BMMs. | CpG inhibits RANKL-induced osteoclastogenesis when added together with RANKL, but stimulates osteoclastogenesis in RANKL-primed cells, even in the absence of RANKL. | CpG down-regulated MCSF receptor when added in the beginning of the culture. The effect of CpG inducing osteoclastogenesis in RANKL primed cells is partially blocked by anti-TNF-α and anti TNFR1 antibodies. | (Zou, Schwartz et al. 2002) |
| Synthetic | CpG-ODN | TLR9 | Mouse osteoblasts/BMMs cocultures | CpG-ODN induce osteoclastogenesis in cocultures of osteoblasts and BMMs | CpG-ODN activates NF-κB and induces p38 and ERK phosphorylation in osteoblasts. CpG-ODN regulates the expression of RANKL in the cocultures, but not in osteoblasts alone, unlike LPS, which directly induces RANKL in osteoblasts. CpG-ODN stimulates IL-1β and TNF-α in osteoclast precursors that indirectly stimulate osteoblasts to produce RANKL and induce osteoclastogenesis. | (Zou, Amcheslavsky et al. 2003) |
| Synthetic | CpG-ODN | TLR9 | Mouse calvarial osteoblasts and BMMs derived from wild type and TLR9^-/-^ mice | CpG-ODN inhibits osteoclatogenesis in early osteoclast precursors, but stimulates osteoclastogenesis in RANKL-primed BMMs.  CpG-ODN marginally up-regulates RANKL in calvarial osteoblasts.  Osteoclasts are not formed in cocultures of TLR^-/-^ osteoblasts and osteoclasts, but when the receptor is present in one or both cells, osteoclasts are formed. | Interaction of CpG-ODN with osteoblasts and osteoclasts is important for modulation of osteoclastogenesis | (Amcheslavsky, Hemmi et al. 2005) |
| Synthetic | CpG-ODN | TLR9 | Primary mouse bone marrow macrophages (BMMs) | CPG-ODN induces IL-12 production and release.  CPG-ODN inhibit RANKL-induced osteoclatogenesis, effect that is inhibited by anti-IL-12 | IL-12 mediates the inhibition of osteoclastogenesis by CpG-ODN | (Amcheslavsky and Bar-Shavit 2006) |
| Synthetic | CpG-ODN | TLR9 | Primary uncommitted BMMs | CPG inhibits osteoclastogenesis by inhibiting c-fos induction by RANKL.  CPG limits ERK phosphorylation (which is upstream to c-fos) by inducing the production of protein phosphatase 2A (PP2A). | CpG-ODN induces PP2A-mediated ERK dephosphorylation limiting c-fos expression that is crucial for osteoclastogeneis. | (Amcheslavsky and Bar-Shavit 2007) |
| Various sources | Peptidoglycan, Poly (I:C) dsRNA, LPS, CpG-ODN | TLR2, 3, 4 and 9 | Mouse BMMs, RAW 264.7 macrophages | All TLR ligands inhibit osteoclast differentiation when added together with RANKL, maintaining the phagocytic capacity. Peptidoglycan and LPS enhance survival of mature osteoclasts. | Preosteoclasts express all TLRs and activation of TLR2, 3 4 and 9 inhibit osteoclastogenesis. Mature osteoclasts express only TLR2 and 4 and that is why only their ligands enhance survival of mature osteoclasts. | (Takami, Kim et al. 2002) |

Abu-Amer, Y., F. P. Ross, J. Edwards and S. L. Teitelbaum (1997). "Lipopolysaccharide-stimulated osteoclastogenesis is mediated by tumor necrosis factor via its P55 receptor." J Clin Invest **100**(6): 1557-1565.

Amano, S., K. Kawakami, H. Iwahashi, S. Kitano and S. Hanazawa (1997). "Functional role of endogenous CD14 in lipopolysaccharide-stimulated bone resorption." J Cell Physiol **173**(3): 301-309.

Amcheslavsky, A. and Z. Bar-Shavit (2006). "Interleukin (IL)-12 mediates the anti-osteoclastogenic activity of CpG-oligodeoxynucleotides." J Cell Physiol **207**(1): 244-250.

Amcheslavsky, A. and Z. Bar-Shavit (2007). "Toll-like receptor 9 ligand blocks osteoclast differentiation through induction of phosphatase." J Bone Miner Res **22**(8): 1301-1310.

Amcheslavsky, A., H. Hemmi, S. Akira and Z. Bar-Shavit (2005). "Differential contribution of osteoclast- and osteoblast-lineage cells to CpG-oligodeoxynucleotide (CpG-ODN) modulation of osteoclastogenesis." J Bone Miner Res **20**(9): 1692-1699.

Chen, Z., L. Su, Q. Xu, J. Katz, S. M. Michalek, M. Fan, X. Feng and P. Zhang (2015). "IL-1R/TLR2 through MyD88 Divergently Modulates Osteoclastogenesis through Regulation of Nuclear Factor of Activated T Cells c1 (NFATc1) and B Lymphocyte-induced Maturation Protein-1 (Blimp1)." J Biol Chem **290**(50): 30163-30174.

Hausmann, E., L. G. Raisz and W. A. Miller (1970). "Endotoxin: stimulation of bone resorption in tissue culture." Science **168**(3933): 862-864.

Hayashi, S., M. Tsuneto, T. Yamada, M. Nose, M. Yoshino, L. D. Shultz and H. Yamazaki (2004). "Lipopolysaccharide-induced osteoclastogenesis in Src homology 2-domain phosphatase-1-deficient viable motheaten mice." Endocrinology **145**(6): 2721-2729.

Horton, J. E., L. G. Raisz, H. A. Simmons, J. J. Oppenheim and S. E. Mergenhagen (1972). "Bone resorbing activity in supernatant fluid from cultured human peripheral blood leukocytes." Science **177**(4051): 793-795.

Ishihara, Y., T. Nishihara, E. Maki, T. Noguchi and T. Koga (1991). "Role of interleukin-1 and prostaglandin in in vitro bone resorption induced by Actinobacillus actinomycetemcomitans lipopolysaccharide." J Periodontal Res **26**(3 Pt 1): 155-160.

Itoh, K., N. Udagawa, K. Kobayashi, K. Suda, X. Li, M. Takami, N. Okahashi, T. Nishihara and N. Takahashi (2003). "Lipopolysaccharide promotes the survival of osteoclasts via Toll-like receptor 4, but cytokine production of osteoclasts in response to lipopolysaccharide is different from that of macrophages." J Immunol **170**(7): 3688-3695.

Ji, J. D., K. H. Park-Min, Z. Shen, R. J. Fajardo, S. R. Goldring, K. P. McHugh and L. B. Ivashkiv (2009). "Inhibition of RANK expression and osteoclastogenesis by TLRs and IFN-gamma in human osteoclast precursors." J Immunol **183**(11): 7223-7233.

Kassem, A., P. Henning, B. Kindlund, C. Lindholm and U. H. Lerner (2015). "TLR5, a novel mediator of innate immunity-induced osteoclastogenesis and bone loss." FASEB J **29**(11): 4449-4460.

Kassem, A., P. Henning, P. Lundberg, P. P. Souza, C. Lindholm and U. H. Lerner (2015). "Porphyromonas gingivalis Stimulates Bone Resorption by Enhancing RANKL (Receptor Activator of NF-kappaB Ligand) through Activation of Toll-like Receptor 2 in Osteoblasts." J Biol Chem **290**(33): 20147-20158.

Kassem, A., C. Lindholm and U. H. Lerner (2016). "Toll-Like Receptor 2 Stimulation of Osteoblasts Mediates Staphylococcus Aureus Induced Bone Resorption and Osteoclastogenesis through Enhanced RANKL." PLoS One **11**(6): e0156708.

Kim, J., J. Yang, O. J. Park, S. S. Kang, W. S. Kim, K. Kurokawa, C. H. Yun, H. H. Kim, B. L. Lee and S. H. Han (2013). "Lipoproteins are an important bacterial component responsible for bone destruction through the induction of osteoclast differentiation and activation." J Bone Miner Res **28**(11): 2381-2391.

Kim, J., J. Yang, O. J. Park, S. S. Kang, C. H. Yun and S. H. Han (2016). "Serum amyloid A inhibits osteoclast differentiation to maintain macrophage function." J Leukoc Biol **99**(4): 595-603.

Kim, K. W., B. M. Kim, J. Y. Won, K. A. Lee, H. R. Kim and S. H. Lee (2019). "Toll-like receptor 7 regulates osteoclastogenesis in rheumatoid arthritis." J Biochem.

Liu, J., S. Wang, P. Zhang, N. Said-Al-Naief, S. M. Michalek and X. Feng (2009). "Molecular mechanism of the bifunctional role of lipopolysaccharide in osteoclastogenesis." J Biol Chem **284**(18): 12512-12523.

Modinger, Y., A. Rapp, J. Pazmandi, A. Vikman, K. Holzmann, M. Haffner-Luntzer, M. Huber-Lang and A. Ignatius (2018). "C5aR1 interacts with TLR2 in osteoblasts and stimulates the osteoclast-inducing chemokine CXCL10." J Cell Mol Med **22**(12): 6002-6014.

Oh, E., H. Y. Lee, H. J. Kim, Y. J. Park, J. K. Seo, J. S. Park and Y. S. Bae (2015). "Serum amyloid A inhibits RANKL-induced osteoclast formation." Exp Mol Med **47**: e194.

Sato, N., N. Takahashi, K. Suda, M. Nakamura, M. Yamaki, T. Ninomiya, Y. Kobayashi, H. Takada, K. Shibata, M. Yamamoto, K. Takeda, S. Akira, T. Noguchi and N. Udagawa (2004). "MyD88 but not TRIF is essential for osteoclastogenesis induced by lipopolysaccharide, diacyl lipopeptide, and IL-1alpha." J Exp Med **200**(5): 601-611.

Takami, M., N. Kim, J. Rho and Y. Choi (2002). "Stimulation by toll-like receptors inhibits osteoclast differentiation." J Immunol **169**(3): 1516-1523.

Yang, J., Y. H. Ryu, C. H. Yun and S. H. Han (2009). "Impaired osteoclastogenesis by staphylococcal lipoteichoic acid through Toll-like receptor 2 with partial involvement of MyD88." J Leukoc Biol **86**(4): 823-831.

Zhang, P., J. Liu, Q. Xu, G. Harber, X. Feng, S. M. Michalek and J. Katz (2011). "TLR2-dependent modulation of osteoclastogenesis by Porphyromonas gingivalis through differential induction of NFATc1 and NF-kappaB." J Biol Chem **286**(27): 24159-24169.

Zou, W., A. Amcheslavsky and Z. Bar-Shavit (2003). "CpG oligodeoxynucleotides modulate the osteoclastogenic activity of osteoblasts via Toll-like receptor 9." J Biol Chem **278**(19): 16732-16740.

Zou, W. and Z. Bar-Shavit (2002). "Dual modulation of osteoclast differentiation by lipopolysaccharide." J Bone Miner Res **17**(7): 1211-1218.

Zou, W., H. Schwartz, S. Endres, G. Hartmann and Z. Bar-Shavit (2002). "CpG oligonucleotides: novel regulators of osteoclast differentiation." FASEB J **16**(3): 274-282.
